# Serum and Urinary N-Terminal Pro-brain Natriuretic Peptides as Biomarkers for Bronchopulmonary Dysplasia of Preterm Neonates

**DOI:** 10.3389/fped.2020.588738

**Published:** 2020-10-28

**Authors:** Zoi Iliodromiti, Evangelos Christou, Nikolaos Vrachnis, Rozeta Sokou, Dionysios Vrachnis, Georgia Mihopoulou, Theodora Boutsikou, Nicoletta Iacovidou

**Affiliations:** ^1^Neonatal Department, Aretaieio Hospital, Medical School of National and Kapodistrian University of Athens, Athens, Greece; ^2^Third Obstetrics and Gynecology Department, Attikon Hospital, Medical School of National and Kapodistrian University of Athens, Athens, Greece; ^3^Molecular and Clinical Sciences Research Institute, St George's University of London, London, United Kingdom; ^4^NICU, “Agios Panteleimon” General Hospital of Nikaia, Piraeus, Greece; ^5^Endocrinology Unit, Second Obstetrics and Gynecology Department, Aretaieio Hospital, Medical School of National and Kapodistrian University of Athens, Athens, Greece; ^6^Second Obstetrics and Gynecology Department, Aretaieio Hospital, Medical School of National and Kapodistrian University of Athens, Athens, Greece

**Keywords:** biomarker, brain, preterm neonate, urinary NTproBNP, serum NTproBNP, bronchopulmonary dysplasia

## Abstract

Bronchopulmonary dysplasia (BPD) is a common cause of respiratory illness in preterm newborns with high morbidity and mortality rates. At present, there are no early prognostic biomarkers that can be used in clinical practice to predict the development of BPD. In this review, we critically appraise evidence regarding the use of serum N-terminal pro-brain natriuretic peptide (NTproBNP) levels as a biomarker for BPD in neonates. Furthermore, we summarize studies assessing the feasibility of urinary NTproBNP levels as a non-invasive method to predict BPD in preterm infants. Multiple studies reported a strong association between NTproBNP serum levels and the onset of BPD. For urinary NTproBNP there is scarce evidence showing an association with BPD. Given the promising data obtained by preliminary studies, further assessment of this biomarker in both serum and urine is needed. Standardized reference values should be defined before conducting any further clinical studies.

## Introduction

Bronchopulmonary dysplasia (BPD) is a common cause of respiratory illness in preterm newborns, with significant morbidity and mortality. The term was introduced by North et al. in 1967 to describe the serious lung condition that arises in preterm infants with respiratory distress syndrome who are in need of mechanical ventilation and high oxygen concentration (1). Since then, various revisions of this definition have been proposed so as to meet the evolving needs. In 2001, the National Institute of Health defined BPD as the use of oxygen supply for 28 days, while it stratified disease severity according to the need for oxygen and/or respiratory support at 36 weeks' postmenstrual age (PMA) or 56 days of age for premature infants at ≥32 weeks' gestational age (2). However, limitations to this definition, due to the improvements in neonatal care, are acknowledged. Therefore, in 2016, the Eunice Kennedy Shriver Institute of Child Health and Health Development (NICHD) proposed a revised definition of BPD in neonates. It specifies that a premature baby, at a gestational age (GA) of <32 weeks, with BPD has radiographically confirmed persistent parenchymal lung disease at a PMA of 36 weeks and requires ventilation techniques to increase FiO_2_ ranges, oxygen levels, or O_2_ concentrations for ≥3 consecutive days to preserve arterial oxygen saturation in the range of 90–95% (3). In 2019, the same Institute classified BPD severity according to the type/modality of respiratory support administered at 36 weeks PMA. This definition adheres to an evidence-based approach rather than expert consensus, excludes the assessment of supplemental oxygen use before and at 36 weeks PMA, and incorporates the long-term outcome of disease that is important for the evolution of new strategies for BPD prevention and treatment (4). In a large retrospective study of 1,914 infants, Tetsuya et al. stated that the use of external oxygen supply at 36 weeks post menstrual age was the most accurate indication to predict severe morbidity at 18–21 months' gestationally corrected age (GCA). Additionally, oxygen use at 40 weeks PMA was significantly correlated to severe respiratory complications between 34 and 44 weeks PMA (5).

These multiple and concurrent definitions of BPD reflect the complexity of the disease. As mortality represents an unusual outcome, pulmonary and neurosensory complications in childhood are determining factors for the characterization of BPD.

The use of antenatal corticosteroids and more advanced ventilation techniques have significantly decreased the incidence of BPD (6, 7). Disease frequency rates are inversely correlated with birth weight. Seventy-five percentage of neonates with BPD are born with an extremely low birth weight (<1,000 g). Although, on average, 20% of neonates in need of mechanical ventilation at birth will develop BPD, disease rates vary among different populations (8). The main factors contributing to BPD are intrauterine growth restriction, antenatal factors, epigenetics, nutritional impairment, various causes of neonatal respiratory distress (meconium aspiration syndrome) (9), hemodynamically-significant patent ductus arteriosus (HsPDA), oxygen toxicity, ventilator-induced lung injury, and postnatal infection. However, the predominant predisposing factor is the degree of prematurity, while the clinical phenotype of the disease varies based on the severity of the parenchymal, airway, and pulmonary vascular disease (3).

The long-term sequelae of BPD include altered pulmonary function, increased incidence of inflammatory lung disease, airway hyperactivity, enhanced infection susceptibility, and development of pulmonary hypertension (PH) (10), as well as developmental delay and cognitive disabilities due to prolonged use of corticosteroids (11). Therefore, early detection and identification of neonates with a high risk of BPD is important. Biomarkers that could detect the disease in an early stage or could identify the population at risk would help design preventative treatment regimens as well as select patients for clinical trials.

To date, no early prognostic biomarker for BPD development is universally used in everyday clinical practice. The aim of this review was to summarize existing data on the use of serum and urine NT-proBNP as a tool for BPD prediction in preterm infants and to assess whether these biomarkers could be correlated to disease progression.

## The Key Role of NT-proBNP

NT-proBNP is secreted by cardiac myocytes in response to volume overload. Brain natriuretic peptide (BNP) is derived from the 134 amino acid precursor pre-proBNP, which is cleaved into an 108-amino acid prohormone proBNP and a 26-amino acid signal peptide (12, 13). Subsequently, proBNP is released into the circulation where the proteases furin (14) and corin (15) cleave it into the physiologically-active hormone BNP (77–108 amino acids) and the inactive metabolite NT-proBNP (1–76 amino acids) (16). NT-proBNP is not biologically active, therefore, it has no active clearance mechanisms; it is a low molecular weight peptide (8.5 kDa) and is removed from plasma via passive excretion by organs with a high blood flow, such as the liver and kidney (17). BNP and NT-proBNP are released into the circulation from the cardiac ventricle at a ratio of 1:1. Plasma proBNP concentrations are higher than that of BNP because proBNP is filtered by the kidneys, unlike BNP, which is metabolized in the systematic circulation via natriuretic peptide receptor-C. Moreover, pro-BNP's half-life is longer than that of BNP (120 vs. 22 min) (18, 19) under normal glomerular filtration rate and it has a more stable *in vitro* chemical structure than BNP. Therefore, proBNP levels in plasma may serve as a reliable biomarker.

There has been increased research interest over the last few years in the use of NT-proBNP as a prognostic biomarker in multiple neonatal diseases, such as diaphragmatic hernia (20), respiratory distress (21, 22), PH (23), and HsPDA (13, 24). Increasing evidence supports the use of serum NT-proBNP levels as a potential biomarker for respiratory morbidities, including BPD in preterm infants (25, 26).

## Serum NTproBNP as a Biomarker for BPD in Neonates

Table 1 summarizes studies conducted to determine the efficacy of serum NT-proBNP levels as a biomarker for screening of early BPD in preterm infants (13, 27–36). A prospective cohort study including 101 extremely low birth weight infants showed that NT-proBNP could be effectively used as a primary prognostic biomarker for BPD development prediction. A remarkably high sensitivity (100%) and high specificity (86%) can be achieved by using cut-off values of 2,264 pg/mL on the 14th postnatal day. Thus, NT-proBNP at 14 days of life (DOL) could be used as an early marker of later BPD development, identifying patients who would benefit from primary, personalized treatment (27). A pilot study including 34 neonates <34 weeks of GA reported that higher serum NT-proBNP levels were associated with an increase in BPD severity at DOL-28 (control group: 1,122 pg/mL vs. BPD group: 3,208 pg/mL). Of the neonates with BPD, 60% were above the cut-off, indicating that premature babies affected by BPD may have very high levels of NT-proBNP, even in the absence of heart disease (31). In 2018, a study including 51 infants born at GA < 30 weeks investigated changes of NT-proBNP values in the 3rd, 10th, 28th, and 26th week of life, as well as the influence of hypoxia, creatinine levels, hemoglobin levels, BPD, and GA on NT-proBNP. On DOL-10, NT-proBNP was a valuable marker for the prediction of severe BPD (13). A retrospective cohort of 147 neonates born at GA of < 32 weeks showed that NT-proBNP levels were higher in those with medium/severe BPD in contrast to those who did not develop BPD or had mild BPD (3,885 vs. 1,259 pg/ml). However, when the biomarker was measured on DOL-1, no correlation between NT-proBNP increase and BPD prognosis was found (32). In agreement with the literature, an observational study with 70 preterm infants revealed that NT-proBNP levels on DOL-28 were significantly higher in those who would later develop moderate to severe BPD, but predictive accuracy was moderate (36).

**Table 1:** Studies on serum NT-proBNP for the assessment of BPD in preterm infants.

**Disease**	**GA**	**Time of life**	**Mean/Median serum NT-proBNP concentration. Control vs. disease group**	**No of patients**	**Year of publication**
BPD	<32 weeks	1–49 days	Day 14: 1.155 vs. 9.707 pg/ml (increased, *p* = 0.0003)	101	2019 (27)
BPD	<30 weeks	3–28 days, 36 weeks	Day 10: cut-off value > 189 pmol/L (84% and specificity 75% predictivity BPD)	51	2018 (13)
BPD	<30 weeks	28, 32, 36 weeks CGA	Week 28 GCA: 500 vs. 1.200 pg/ml Week 32 GCA: 608 vs. 1.564 pg/ml Week 36 GCA: 918 vs. 1.840 pg/ml (increased, *p* < 0.01)	44	2018 (28)
BPD	<32 weeks	48–72 h	15.578 vs. 52.866 pg/ml (increased, *p* = 0.000)	117	2017 (29)
BPD	<32 weeks	3 days	3.495 vs. 11.607 ng/l (increased, *p* < 0.001)	134	2015 (30)
BPD	<34 weeks	4 weeks	1.122 vs. 3.208 pg/ml (increased, *p* = 0.006)	36	2010 (31)
BPD	<32 weeks	1 day	1.259 vs. 3.855 pg/ml (increased, *p* < 0.001)	147	2019 (32)
BPD	<32 weeks	5–10 days	(1.427) vs. 8.849 ng/L (increased, *p* < 0.001)	110	2018 (33)
BPD	30 ± 2.9 Mean SD (wk)	9.8 ± 2.3 Mean SD (mo)	No prediction of BPD	28	2013 (34)
BPD-PH	<27 weeks or/and <750 g	36–38 weeks CGA	>1.000 pg/ml (increased, *p* < 0.001)	20	2016 (35)
BPD	<30 weeks	28 postnatal day	Group 2 (moderate BPD) vs. Group 1 (mild BPD): 845 vs. 726 pg/ml (increased, *p* = 0.02)	70	2019 (36)

*GA, gestational age; BPD, bronchopulmonary dysplasia; GCA, corrected gestational age; PH, pulmonary hypertension; SD, standard deviation; wk, week; mo, months*.

## Serum NT-proBNP as an Alternative Indicator for Detection of Secondary Pulmonary Hypertension Due to BPD

Alterations in the distribution of pulmonary vessels in patients with severe BDP lead to increased pulmonary vascular resistance and PH (10, 37). Approximately 18–25% of premature infants who have moderate or severe BPD will develop PH (BPD-PH) (38, 39), which is associated with significant mortality (40, 41). PH, defined as a mean pulmonary artery pressure of >25 mmHg at 3 months of life (38), is diagnosed by measuring pulmonary artery pressure during cardiac catheterization. However, this may not always be feasible in neonates (42). Although Doppler echocardiography measurements may be used to diagnose PH, they are often inaccurate and there is a lack of specific guidelines for diagnosing PH in neonates (43). This deficiency has led to the proposal for the use of several biomarkers, including NT-proBNP, to diagnose BPD-PH in premature infants.

A recent observational study reported serial serum NT-proBNP levels and echocardiography parameters at 28, 32, and 36 weeks GCA. The authors observed a correlation between NT-proBNP cut-off levels at 28 weeks GCA and the development of BPD (578,1 pg/ml) and BPD-PH (2,329 pg/ml) at 36 weeks GCA (28). These results are in line with those of a previous study in 20 preterm infants <27 weeks GCA and with a birth weight of <750 g. In this study, echocardiography was performed at 36–38 weeks GCA and the concentrations of NT-proBNP in serum in the first week of life were assessed. Infants in the BPD-PH group had significantly higher levels of NT-proBNP than the control group (BPD-PH group 1,650 pg/mL vs. control group: 520 pg/mL) (35).

The occurrence of secondary PH is an increasingly recognized complication of BPD. Thus, the role of serial measurements of NT-proBNP in infants with secondary PH due to BPD should be identified in future studies. In addition, variability in NT-proBNP levels in response to different pharmaceutical agents for secondary PH due to BPD, such as sildenafil, epoprostenol, and inhaled nitric oxide, should be assessed to determine whether it can be used for monitoring disease progress.

## Urinary NTproBNP as a Biomarker for BPD in Neonates

Urinary NTproBNP levels have also been studied in premature infants to predict early BPD (24). The intraindividual variation of urinary biomarker precludes its use as a biomarker in adults (44). However, several studies reported an association between urinary NT-proBNP and neonatal morbidities, such as HsPDA and retinopathy of prematurity (45–48).

The clearance mechanism of NT-proBNP in preterm infants is currently poorly understood. On the other hand, the higher stability and lower clearance rate of NT-proBNP compared to BNP (the latter due to less accessible clearance receptors) clearly indicate its potential use as a biomarker.

Czernik et al. studied 136 preterm infants with a median GA of 28 days and determined urinary NT-proBNP levels on postnatal DOL-2,−7,−14, and −28. They found a significant association between BPD and urine NT-proBNP normalized to creatinine on DOL-7 (45).

In a pilot study of 54 premature infants, NT-proBNP measurement was found to be feasible and may serve as a reliable noninvasive screening test for BPD-PH in neonates. The biomarker levels were significantly higher in the BPD-PH group than those in the control group. In addition, cutoff values of 2,345, 206.50, and 23.90 pg/ml at 28, 32, and 36 weeks of GA, respectively, resulted in a >80% of sensitivity for PH (49).

Therefore, urinary NT-proBNP may have potential as a non-invasive biomarker, although further large-scale studies need to be conducted to confirm these initial results.

## Future Developments in the Field

Evidence for the use of NT-proBNP as a biomarker for BPD has some limitations at present. All studies included in this review are either retrospective or observational studies. Moreover, the sample size of most of the reported observational studies was small and these studies also present the risk of bias due to unmeasured confounders. Again, we should highlight the need for large, multicenter studies. Multiple BPD definitions and severity classifications set limits concerning the comparability of samples among studies. In addition, levels of NT-proBNP detected in blood or urine are dependent on the assay used to determine them. Possible variations in laboratory techniques used for NT-proBNP measurement should also be taken into consideration. Every kit establishes specific normal limits given that a standardized reference value is not as yet available. Hence, future studies should define reference values so as to better compare study results and assess the validity of this biomarker. Additionally, randomized clinical trials are warranted to evaluate the cost-effectiveness of the use of NT-proBNP as a biomarker for BPD in neonates.

## Discussion

In adults, BNP and NT-proBNP have emerged as powerful biomarkers for the prognosis and outcome prediction of various cardiovascular disorders, including PH, congestive heart failure, and respiratory distress (50).

BNP is not transferred to the fetus from the maternal circulation (51) and an acute postpartum BNP increase is observed among neonates, followed by gradual stabilization in the 3rd month of life. In cases of sepsis, renal injury, PH, HsDPA, and congestive heart defects with increased heart volume NT-proBNP remains increased (52). On the other hand, upregulation of natriuretic peptide synthesis in the ventricular myocardium is observed in conditions of sustained ventricular blood volume and pressure overload. The correlation between hsPDA and BPD development, as well as the pulmonary overcirculation and hemodynamic overflow observed in BPD, are factors that may lead to elevated NT-proBNP levels. High pulmonary vasculature flow in HsPDA and other perinatal conditions, such as oxidative stress, chorioamnionitis, intrauterine growth delay, and ventilator-induced lung injury, affect pulmonary vascular development and tone and result in an increase of the right ventricular afterload and enhancement of natriuretic peptide secretion (29). As a consequence of the strong correlation between BPD and diastolic dysfunction and abnormal left ventricle myocardial performance index in preterm infants (53), there is also a significant association between NT-proBNP levels and the onset of BPD.

Although various predictive models of BPD risk have been published, their utilization in daily clinical practice remains uncommon. The development of non-invasive ventilation as the main modality of respiratory support in the delivery room has changed the long-term outcomes of the disease.

The traditional approach included risk factors such as lower birth weight, lower gestational age, male sex, HsPDA, sepsis, and mechanical ventilation, among many others (54). However, this approach was not based on postnatal age; therefore, it was not possible to evaluate the multiple effects of neonatal exposures over time.

Nowadays, it is accepted that the most important factor to eliminate the incidence and severity of BPD is avoidance of intubation and mechanical ventilation (55). Moreover, novel methods of non-invasive ventilation and surfactant delivery have improved long-term outcomes. Nevertheless, it is important to focus research interest on the discovery of novel biomarkers with improved accuracy, enhanced measurability, and wider applicability (56).

There is a growing body of evidence showing the applicability of serum NT-proBNP as a marker in the diagnosis and prognosis of respiratory complications in preterm infants. Supporting this hypothesis, the studies included in this review have shown a strong association between increased serum levels of NT-proBNP in preterm infants and the occurrence of BPD.

In neonates, measurements of biological markers in a peripheral smear are limited by the small volumes collected. However, urinary NT-proBNP is easier to sample, allows repetition measurements, and has a lower cost (57, 58) than assessments in the blood (59). In several studies, urinary NT-proBNP has also been shown to predict HsPDA in the immediate postnatal period. Consequently, the measurement of urinary NT-proBNP as a non-invasive and potent method to predict the development of BPD or cardiovascular diseases in neonates shows considerable promise.

## Conclusion

Serum NT-proBNP may be a reliable biomarker in predicting outcomes in preterm infants who are at high risk of developing BPD. The measurement of NT-proBNP in urine samples is a promising, non-invasive, and reliable method. However, it remains poorly studied; thus, further studies of urine NT-proBNP measurement for the prediction or early detection of severe BPD are warranted. Furthermore, there is an urgent need to standardize NT-proBNP measurements and reference values.

## Author Contributions

ZI and EC conceptualized the study, coordinated and supervised data collection, carried out elements of data analysis, and wrote the manuscript. NV, TB, and NI designed the study and reviewed and revised the manuscript. RS and DV provided technical advice and wrote parts of the manuscript. GM assisted in the data collection and reviewed the manuscript. All authors approved the final manuscript as submitted and agree to be accountable for all aspects of the work.

## Conflict of Interest

The authors declare that the research was conducted in the absence of any commercial or financial relationships that could be construed as a potential conflict of interest.

## Abbreviations

BPD: Bronchopulmonary dysplasia
NTproBNP: N-terminal pro-brain natriuretic peptide
BNP: brain natriuretic peptide
PMA: post menstrual age
GA: gestational age
HsPDA: hemodynamically-significant patent ductus arteriosus
PH: pulmonary hypertension
DOL: days of life
GCA: gestationally corrected age.
